# "The Hospital" Medical Book Supplement.—No. XII

**Published:** 1908-11-21

**Authors:** 


### "THE HOSPITAL" MEDICAL BOOK SUPPLEMENT.—No. XII.

CONTENTS.

**Pædiatrics—**
Tuberculosis in Infancy and Childhood ... 199
Diet in Infancy ... 199
How to Nurse Sick Children ... 199
**Therapeutics—**
Therapeutics of the Circulation ... 200
A Manual of Natural Therapy ... 200
**Diseases of the Heart—**
Diseases of the Heart ... 200
**Otology—**
A Text-book of Diseases of the Ear ... 201
**Surgery—**
When to Operate in Inflammation of the Appendix ... 201
A Synopsis of Surgery ... 201
Pain: Its Causation and Diagnostic Significance in Internal Diseases ... 201
**Miscellaneous Items—**
The Sexual Life of Our Time, in its Relation to Modern Civilisation ... 202
The Letters of Queen Victoria ... 202
The New Zealand Shipping Company's Pocket Book ... 202

### PÆDIATRICS.

Tuberculosis in Infancy and Childhood. By Various Writers. Edited by T. N. Kelynack, M.D. Pp. xiii. and 376; 27 illustrations. (London: Baillière, Tindall, and Cox. Price 12s. 6d. net.)

This polygraph on tuberculosis consists of the contributions of thirty-eight writers and an introduction by the Editor. The contributors are drawn from almost all parts of the world and hold recognised positions as authorities on tuberculous questions or on the particular specialty on which they write. Consequently it is impossible in a short review to refer to the individual merits of each essay. It is difficult, if not impossible, even under the most strict editorship, to prevent overlapping and repetition in the discussion of some of the more important features in the problem of tuberculosis. Perhaps this is an advantage, inasmuch as one is put in possession of the different views held and the arguments in support thereof. There is a certain amount of repetition of somewhat dreary statistics which might have been collated in a separate chapter for reference. It would, in our opinion, have been advantageous to have given a brief summary of the conclusions arrived at by the English Royal Commission and of the German Imperial Commission on Tuberculosis, rather than to permit the different contributors to include in their comments only those in support of their particular views. As an instance of repetition, Dr. R. A. Young, of London, writes on intrathoracic tuberculosis and includes the pulmonary types, and is immediately followed by Dr. Emmet Holt, of New York, with an article on pulmonary tuberculosis in which he includes the affections of the thoracic glands. Holt insists on the frequency of pulmonary tuberculosis in infancy and regards the lungs as the most frequent clinical seat of the disease at all ages. He attaches a great deal more importance to human than to milk infection. Dr. J. S. Fowler ascribes one-fourth of the cases to milk infection and advocates pasteurisation or sterilisation of milk in spite of its known disadvantages. Dr. Nathan Raw regards primary pulmonary tuberculosis as due to inhalation and other forms as of alimentary origin. He advocates the view that the human bacillus can cause primary intestinal ulceration, whereas the bovine bacillus passes through the mucosa and sets up glandular tuberculosis. Apparently he ascribes intestinal ulcers to local infection by ingested bacilli and not to a blood or lymphatic infection. He holds that the human and bovine bacilli are different varieties of the same species, but antagonistic to each other, and mutually protective; and that mild bovine tuberculosis protects against phthisis. If these views were accepted it might be a wise precaution to feed all infants on tuberculous cow's milk as a protective measure. Another statement to which we take exception is that of Dr. J. McCarthy, of Pennsylvania, that tuberculous meningitis is rare during the first and second year of life. It certainly is not so in this country. Thus, of 71 cases under 12 years, 54 were under 5 years, and of these 10 were in the first and 16 in the second year of life (Cautley, 1907). These are minor criticisms. The work as a whole is most valuable. It contains the most recent views on this disease, and, in addition, useful chapters on its frequency in other countries and the methods adopted for its prevention, diagnosis, and cure.

Diet in Infancy. By A. Dingwall-Fordyce, M.D., F.R.C.P.Ed. (London: W. Green and Sons. 1908. Pp. 174. Price 3s. 6d. net.)

In putting before the profession this volume the author, in his prefatory note, strikes a note which is echoed throughout the text. He pretends, he says, neither to heights of eloquence nor to depths of scientific information, but aims merely at practical utility. This claim is one which can be endorsed. The arrangement of the subject matter is good, the language precise and easy to follow, and a healthy tone of common sense pervades the pages throughout. Dr. Dingwall-Fordyce is evidently no faddist, and he wisely relegates to appendices some complicated tables and statistics which would interfere considerably with the simplicity and directness of the chapters on infant feeding itself. In the first chapter, which comprises general considerations concerning the healthy and the sick infant, italics are allowed to run riot to an extent which entirely spoils their effect; but on settling down to a description of normal infancy, mother's milk, etc., in the next chapter, the author improves in this respect. His tone throughout is dogmatic, a plan which always pleases readers as long as they are in agreement with the sentiments expressed, but is distasteful to those who are not. Luckily in this case we are in accord with nearly everything which is set down, and we are sure an addition of value has been made to the already copious literature on this subject.

How to Nurse Sick Children. By the late Charles West, M.D. (London: Longmans, Green and Co. Price 1s. net.)

The reissue of this very old work by the founder of the Great Ormond Street Hospital for Sick Children is in our opinion a mistake. However valuable it may have been thirty years ago, the times have changed since then. In its original (and present) form the monograph is too diffuse and not nearly complete from the point of view of the trained nurse, whereas it is too technical for the nursemaid or the parent of a sick child. There is, of course, much in it of importance and of truth about the nursing of sick children the utility of which time can never efface; but while there are lessons that the most experienced need not be ashamed to take to heart from its pages, it is not a publication that nowadays one is conscious of any niche for on the shelves of medical literature.

### THERAPEUTICS.

Therapeutics of the Circulation. By Sir Lauder Brunton, Bart., M.D., F.R.S. (London: Published under the auspices of the University of London by John Murray. 1908. Pp. xii. + 272. Figs. 235. Price 7s. 6d. net.)

This volume comprises the subject matter of eight lectures delivered in the spring of 1905 in the Physiological Laboratory of the University of London by the author. It is well printed, elegantly bound, profusely illustrated, and indexed in a way that is an example to the majority of modern books. The figures, diagrams, and other illustrations are all remarkably clear and good. The weight of paper required to ensure this has made the volume unavoidably heavy. The various instruments that have been devised for measuring the blood-pressure clinically, as well as certain modifications devised by the author, will be of particular interest to all practitioners. The scope of the lectures upon which the book is founded is in accordance with the general purpose expressed by the University in the establishment of the Physiological Laboratory, namely, "to present the results of recent investigations by the investigators themselves, orally and with experimental demonstration, in the lecture-room and outside the lecture-room, by monographs approved by the University." The work embodies a very large number of physiological experiments, but we would draw particular attention to the fact that the author treats them throughout from the point of view of the bedside physician. There are very valuable suggestions as to treatment, based upon applied physiology. It might be thought that a book upon the heart and circulation issued in the year 1908 could not but contain the gist of recent work upon venous pulse-tracings upon which so many observations have been made by Dr. James Mackenzie, and by Wenckebach on the Continent. This is not the case. The explanation of this is that the lectures were delivered in 1905, and although their issue in book form was unavoidably delayed for three years it was thought best to leave the lectures upon the whole in their original form, adding a few remarks to them in the form of appendices.

A Manual of Natural Therapy. By Thomas D. Luke, M.D., F.R.C.S. (Edin.). Pp. xvi. + 303, with 30 plates and 125 illustrations. (Bristol: John Wright and Sons, Ltd. Price 7s. 6d. net.)

This manual deals fully and professionally with methods of medical treatment other than by drugs. It is extremely difficult, in dealing with special forms of treatment of this nature, not to be carried away into enthusiastic extremes or into some approach to charlatanism. The author seems to have avoided this successfully; and although there are bound to be different opinions as to the exact value of the forms of treatment described, and as to the interpretation of their effects, we think that he has produced a book that will be most useful to all medical men, especially to general practitioners and to consulting physicians. The letterpress is in itself clear and interesting, and it is made the more so by reason of the many illustrations. The work is dedicated to Wilhelm W. Winternitz, M.D., who was the first to develop hydrotherapy on scientific and physiological lines, with truly remarkable results, and who has been appointed to a special Chair of Hydrotherapeutics in the University of Vienna. It is often complained that English doctors take less care in regard to the details of such treatment as the book deals with than Continental doctors do. In some cases this is an advantage—the treatment can be overdone. In other cases, on the other hand, it is a decided disadvantage, and there can be no doubt that part of the lack of detail is due to lack of knowledge. The volume before us gives concise and yet perfectly clear accounts, not only of hydrotherapeutics, electro-therapeutics, and so forth in a general way, but also of each different process that may be required.

### DISEASES OF THE HEART.

Diseases of the Heart. By James Mackenzie, M.D., M.R.C.P. Pp. xx. + 386, with 5 plates and 268 figures. (Oxford Medical Publications. Henry Frowde, and Hodder and Stoughton. Price 25s. net.)

Monographs upon the heart and circulatory system seem to be an abundant crop this year. Practitioners may well be at a loss to know in what respects each work differs from the others, and which book to purchase for any particular line of information. The book before us has the immense advantage of being the outcome of the personal observations and experiences of an original worker, himself a busy practitioner, who has attacked the question of heart failure upon lines which throw not only new light, but an absolutely new kind of light, upon it. The author's researches upon the nature and varieties of cardiac irregularity have brought him world-wide fame. Wenckebach upon the Continent, and Mackenzie in this country were, we believe, the independent originators of routine investigations of venous pulse tracings in all heart-cases. By the steady accumulation of thousands of graphic records, taken simultaneously from the radial pulse, the jugular vein, the præcordial region, and, perhaps, from the liver or elsewhere too, and by the most careful, critical, and withal clear-sighted comparison of one tracing with another, Dr. Mackenzie has been able to elucidate a great many things about heart complaints that were formerly unexplainable. It is particularly in connection with cardiac irregularities that discoveries of great moment have been made. Students of medicine interest themselves less in pulse tracings now than formerly. It will be a revelation to many that so much has been learned from them, and that it seems likely that there is much more to learn. The Mackenzie clinical polygraph is an instrument to be widely used. At the same time the busy practitioner will be glad to be reassured that "in routine practice it is not usually necessary to take graphic records. If one is trained to make careful and minute observations by the ordinary methods, and to have these checked by graphic records, one can ultimately acquire the power of recognising the majority of movements of the circulation without graphic records." Dr. Mackenzie gives us the gist of what his own graphic records have taught him. His teaching needs a clear head to follow it, but when followed, and whilst it is being followed, it gives one a far wider view of abnormal cardiac phenomena than was in any way possible before. Some of the greatest of modern advances in our knowledge of cardiac affections are due to Dr. Mackenzie. We would add that besides what may seem to be rather abstruse discussions there are very practical suggestions as to treatment. It is a matter for congratulation that clinical work of such high calibre is still being done in Great Britain.

### SURGERY.

When to Operate in Inflammation of the Appendix. By C. Mansell Moullin, M.D., F.R.C.S Third Edition, 1908. (London: Bale, Sons, and Danielsson. Price 2s. 6d. net.)

It is a curious thing that after years of discussion the broad principles of correct treatment for so common and so serious a disease as appendicitis are not yet definitely settled. Mr. Moullin is well known as one of the forward school in this matter; but although the writings and contentions of this school are now long familiar, their acceptance is still somewhat partial, or at least not universal. In this latest edition of a monograph which has already secured ample recognition as a weighty contribution to the study of appendicitis, the author has revised and partly rewritten much of it. As an exposition of Mr. Moullin's (and very many other surgeons') views on the subject in hand, these lectures are admirably clear and concise. The author does well to emphasise the fact that fulminating cases are nearly always primary, and the reason is doubtless, as he suggests, the lack of adhesions dating from previous attacks. We confess to a slight scepticism on certain points dealt with, but taken as a whole this republication is one we heartily welcome.

A Synopsis of Surgery. By Ernest W. Hey Groves, M.D., M.S., F.R.C.S. (Bristol: John Wright and Sons, Ltd. Price 7s. 6d. net.)

This is a useful and well-compiled summary of the essentials of surgery—a condensation, in fact, of the information that is usually spread over many pages in ordinary surgical text-books. The author has added greatly to the usefulness of the book by consulting special monographs and by including a list of "surface markings" at the end. The text is singularly free from errors, typographical or otherwise, and there are few statements at which the critic can cavil. Books of this kind, intended for students preparing for examination, must be dogmatic. Still, we would not care to impress even upon an elementary student the opinion that rodent ulcer contains no cell nests. Nor would we care to use the word "traumatism" in the sense that the compiler does. But these are minor details, and the book is certainly to be commended to the student who wishes to refresh his mind on essentials. There appears to be a demand for such compilations, and this is one of the best of its kind we have seen.

Pain: Its Causation and Diagnostic Significance in Internal Diseases. By Rudolph Schmidt; translated and edited by Karl M. Vogel, M.D., and Hans Zinsser, A.M., M.D. (London: T. Fisher Unwin. 1908. Pp. 326. Price 12s. 6d. net.)

It is always a matter of the greatest difficulty to educate oneself to be able to form an adequate mental picture of subjective symptoms. Bruits that one can hear, lumps that one can feel, are very much easier to be sure about than are pains which the patient can merely describe. Whenever there are objective symptoms or signs diagnosis is materially assisted, and the trend of modern medical research is constantly towards objective things such as skiagrams and serum reactions. The result is that amongst the younger generation of medical men, even amongst those who have a good command of the methods of objective examination, there is a great deficiency in the ability to make use of the information conveyed by the manifestations of pain. In many cases too little care is bestowed in accurately locating the pain and analysing its characteristics—such as its time-relations, its directions of radiation, the effects upon it of posture, motion, pressure, food, drugs, sleep, and so forth. The subject is an extremely difficult one, being greatly complicated by individual variability. Nevertheless there cannot be a doubt but that to be a good clinician one must pay a careful consideration to the patient's own sensations. If the book before us does no more than emphasise this it will have done good work. Older practitioners who are familiar with Hilton's "Rest and Pain" will need no newer book; younger men would do well to read Schmidt's work. It is impossible to learn all about the interpretations of pains except by actual practice, but a book upon the subject indicates the broad lines upon which observations should be made. The one before us bears the stamp of personal experience throughout. It surprises us that the original German edition makes no mention of "Head's Areas," which are so familiar in English-speaking countries. This has been corrected by the addition of a chapter upon "Cutaneous Tenderness in Visceral Disease" by the translators, together with clear diagrams of areas of pain or tenderness in disease.

### OTOLOGY.

A Text-book of Diseases of the Ear. By Macleod Yearsley, F.R.C.S. (London: Kegan Paul, Trench, Trübner and Co. 1908. Octavo, pp. 452 + xi. 128 illustrations. Price 18s. net.)

In this volume, which is stated in the preface to be an enlargement of the author's smaller manual "Common Diseases of the Ear," Mr. Yearsley has given a very thorough and conscientious account, not only of the common but of the more unusual ear diseases. It is conspicuously up-to-date, especially in those parts dealing with operations on the internal ear; the chapter on otosclerosis, also, is particularly clear and complete. The author very wisely makes no attempt to subdivide clinically the early stages of the acute inflammations of the middle-ear into suppurative and non-suppurative, and he alludes in strong terms to the amount of preventable ear disease which is even now allowed to occur unchecked for want of proper attention, especially during the course of the infectious fevers. We welcome the expression of the opinion, now generally held by otologists, that the cerebral complications of aural suppuration should always be attacked by the mastoid route, and the consequent omission of descriptions of trephining operations for cerebral and cerebellar abscesses. Chapter XII., which gives a full and judicious description of the aural complications of general diseases, should prove most useful to the general physician, whilst a very complete series of references will be found of value to the specialist. The illustrations, many from original photographs, are good, but some are printed too deeply, and two—figs. 6 and 127—are wrongly placed. There are many figures of instruments—perhaps too many—and we venture to suggest that illustrations of such instruments as a mallet, a periosteal elevator, and a forehead mirror, are waste of space. With regard to the latter, the error which so frequently appears in text-books is repeated; the focal length of the ordinary mirror is not 14, but 7 to 7½ inches for parallel rays. Although in the later chapters lucidity is in a few places sacrificed to compression, the book is well and clearly written and can be recommended as a most satisfactory text-book for practitioners.

### MISCELLANEOUS ITEMS.

THE SEXUAL LIFE OF OUR TIME, IN ITS RELATION TO MODERN CIVILISATION. By IWAN BLOCH, M.D. Translated from the Sixth German Edition by M. Eden Paul, M.D. (London : Rebman, Ltd. Price 21s. net.)

IT is impossible not to recognise the sincerity and earnestness of spirit with which Dr. Bloch has written this comprehensive and impartial study of a difficult question. If we cannot endorse his views we can nevertheless appreciate his desire to pass "through error to truth," and through evil to good, even if the truth be somewhat unpalatable and the good somewhat obscure. We are not, however, entirely convinced of the value of this work to anyone who is not within the small circle of alienists and specialists in sexual pathology. The general practitioner will very rarely find that such a detailed exposition will be of much use to him. For the general public, to whom the translator addresses a prefatory notice, we consider that only certain chapters will be useful. Even here we are rather afraid that the book may produce the exactly opposite effect to that desired by the author. It is our opinion that the book as a whole is entirely unsuitable for general reading. Though we recognise fully his clever psychology of love, and his masterly handling of many difficult questions, we must deprecate the inclusion of elaborate detailed accounts of cases of psychopathia sexualis. We are fully aware of the objection which will be made to the exclusion of any part of a book whose scope is avowedly encyclopædic. We know well that "thoroughness" is the first requisite in a scientific work of this kind, and Dr. Bloch is eminently thorough. But we fail to see of what scientific interest or practical value the greater part of this book can be to the general public or to more than a small minority of medical men, and even for this small minority we think that a great deal of the book could have been omitted without the loss of anything really essential. In conclusion we may say that even in his most idealist chapter on "Free Love," Dr. Bloch is entirely on the side of morality. We wish that it were possible to publish some of the more general chapters separately in book form. There is much that is interesting and valuable in Dr. Bloch's monumental work, but we must repeat our opinion that anything but good is likely to come of its circulation among laymen.

THE LETTERS OF QUEEN VICTORIA : A Selection from Her Majesty's Correspondence between the years 1837 and 1861. Published by command of His Majesty the King. Edited by ARTHUR CHRISTOPHER BENSON, M.A., and VISCOUNT ESHER, G.C.V.O., K.C.B. In three volumes. Crown 8vo. Pp. 1504. With 16 illustrations. Price 6s. net the set. (London : John Murray.)

THE publication of "The Letters of Queen Victoria" in a popular edition of three small volumes, at a price within the reach of almost every book-lover and student of modern history, must be regarded as a public benefaction. Merely from the standpoint of literature these three volumes are of considerable value and interest; but, quite apart from this, they throw an unusually clear and reliable light upon national affairs of the highest importance; and, furthermore, throughout the whole work there runs a deeply interesting personal note, which would rivet the reader's attention, even if the personality revealed were of far less public significance than that of her late Majesty. This issue contains the complete text of the original edition, which, as is well known, was published at a price far beyond the means of the ordinary reading public. The red binding of these three volumes is simple and tasteful, the printing is admirably clear, and the text shows signs of careful revision. In addition to these advantages, there are sixteen reproductions of portraits, and a full and accurate index. The editors have used great skill and judgment in their selections from Queen Victoria's vast public and private correspondence, and under their discriminating guidance the reader may gain a remarkably clear insight into the development of her Majesty's character during the first twenty-four years of her reign. Many will learn here for the first time of the close and authoritative grasp which the Queen held upon all public matters, whether of home or of foreign policy, and will catch interesting and suggestive glimpses of her personal feelings towards those whom she thus honoured with her correspondence. The editors state that their aim, throughout the task of selection, has been "to produce a book for British citizens and British subjects, rather than a book for students of political history": and in this they have succeeded admirably. We may note that scattered here and there in the Queen's letters are many affectionate references to her medical attendants, especially to Sir James Clark and Sir William Jenner.

THE NEW ZEALAND SHIPPING COMPANY'S POCKET BOOK. (London : A. and C. Black. Price 2s. 6d.)

OF all oversea colonies, New Zealand has always been one to stimulate in the highest degree the interest of stay-at-home Englishmen. Its situation is more nearly the counterpart in the Southern hemisphere of our own in the Northern than that of any other part of the Empire; its inhabitants are practically of pure British blood; its products rival those of our own farmers in our own markets; its athletes can teach us how to play our own games. In fact, New Zealand is more nearly Britain-overseas than any other of our possessions. For anyone contemplating a visit to these Islands of the Blest a more useful and convenient handbook than that issued by the great firm of mail steamship owners it would be difficult to devise. The account of New Zealand itself is by the Hon. W. P. Reeves, for many years the Crown Agent of the Colony in this country, with chapters on individual towns and districts by prominent local authors. There are numerous illustrations, beautifully reproduced in colours, which add very greatly to the attractiveness already lent by interesting text, good paper, printing, and general "get up."

